# Healthcare Research in Mass Religious Gatherings and Emergency Management: A Comprehensive Narrative Review

**DOI:** 10.3390/healthcare11020244

**Published:** 2023-01-13

**Authors:** Mater Almehmadi, Jaber S. Alqahtani

**Affiliations:** 1 UCL Institute for Risk and Disaster, University College London, London WC1E 6BT, UK; 2 Department of Respiratory Care, Prince Sultan Military College of Health Sciences, Dammam 34313, Saudi Arabia

**Keywords:** mass, religious, gatherings, research, Hajj, infectious diseases

## Abstract

Religious mass gatherings, especially pilgrimages of various faiths, involve overcrowding and the international movement of people, exposing individuals to significant health risks, such as the spread of infectious diseases, crowds, exposure to bad weather, physical stress, or risks due to pre-existing medical conditions. This paper aims to review the literature related to health care research on religious mass gatherings, with special reference to the role of awareness creation, training, and risk awareness for individuals during Hajj. The results indicated that the research on health risks associated with large-scale gatherings showed that some countries (which witness religious gatherings) follow effective preventive measures to reduce health risks, while some countries did not (and linked this to its poor infrastructure and the low standard of living in it, such as India). It also showed that most studies overlooked identifying the causes of infectious diseases and determining the perceptions of participants in mass gatherings. While it showed that environmental factors strongly influence the emergence of infectious diseases among individuals, the results also showed the scarcity of research that revolves around the awareness of community members, the health risks of mass gatherings, preventive measures against diseases, and the main effects on individuals’ perceptions of risks. The results also showed a lack of research evidence on how pilgrims perceive risks, adopt information, and interact with their willingness to be trained in preventive measures.

## 1. Introduction

Healthcare is one of the essential elements to consider in planning for mass gatherings. Even in events in which everything is managed smoothly, it has been noted that approximately 1.5% of people will require medical assistance, associated both with different kind of ‘physical stress’ or ‘pre-existing’ medical conditions [1]. These include, for example, exposure to bad weather and injuries from crowd stampedes. Mass gatherings are becoming particularly relevant as vectors for possible epidemic viral diseases in which the event may help the virus to mutate, as in the case of avian influenza [2]. Crowded spaces, coupled with the stress and fatigue that characterize them, can create a suitable environment for the spread of infectious diseases [3]. This presents different challenges for analysis. A large amount of the state-of-the-art explained how viruses spread and the physical dynamics of spreading, such as in case of influenzas or allergy [4], but this has not been consistently translated in the academic literature on disaster risk reduction, until recently [5].

While the concept of mass gathering medicine is not new, it has especially become a topic of discussion in recent years. According to Alrabeeah et al. [6], mass gathering medicine can be defined as a field of study within medicine that deals with the public and private health risks and the health impact of mass gathering events. Mass gathering medicine has been in effect for decades through the delivery of health services at mass gathering events. However, it was only formalized as a field of study within medicine by the 2010 Jeddah declaration. The World Health Assembly of Ministers of Health then lend greater legitimacy to the field by adopting it as a formal discipline in 2014 [5]. Mass gathering medicine deals with a wealth of health risks associated with these events. Some of these risks include infectious illnesses, non-infectious illnesses, injury, environmentally related illness, and illnesses/injuries related to deliberate acts [6]. Given this variety of health risks, mass gathering medicine is described as very diverse. This, combined with it only being emergent as a formal medical discipline, have contributed numerous knowledge gap areas that require further exploration. That said, there is a consensus that much of this knowledge can be gained by investigating the large and frequent religious mass gathering events such as the Hajj and the Kumbh Mela.

Disaster risk in mass gathering events is typically from hazards interacting with existing individual vulnerabilities, and much of the current research is concentrated on the transmission of travel-related infectious diseases [3]. Moreover, Alexander highlighted that planning pandemics need to consider both the medical and non-medical nature of the issue, including factors such as the implications of a globalized society and global transportation networks in facilitating the spreading of the infection [1]. Some significant steps for improving the situation have been undertaken within the Sendai Framework for Disaster Risk Reduction (SFDRR). The SFDRR for disaster risk reduction was adopted as a non-binding agreement by the United Nation’s member states in 2015, and it attributes a great deal of importance to improving the resilience of public healthcare including in mass gatherings [7]. A significant portion of the research into mass gathering events has concentrated on global sporting events. Events such as the Olympics and the World Cup not only occur frequently and attract millions of visitors, but they are also at different locations, thus allowing for the collection of varying insights. According to the CDC, the assessment of mass gathering events can effectively be carried out by looking at characteristics related to “location, venue, purpose, size, participants, duration, timing, activities, and capacity” [8]. The location is crucial as different countries have different levels of infrastructure development, health regulations, climates, and security arrangements, all of which can affect the health risks posed to visitors.

As the current literature continues to expand and its relevance increases, there is a strong need to define the existing gaps in research and the areas in need of strengthening to improve practices associated with the implementation of the SFDRR. There is a need to focus on the physical dynamics at mass gatherings and how they influence the spreading of contagious illnesses. Additionally, the role played by awareness, training, and risk perception in the spreading of these illnesses also needs to be defined.

This review focuses on a specific category of mass gatherings that are particularly extensive for their participation and are concentrated on specific geographical locations: religious events. Most people around the world profess religions, according to a report issued by the Pew Research Center, in 2012. It showed that the global religious scene contains more than 5.8 billion people professing religions. The religious diversity is panoramic, and includes three main religious sects: Christianity, Islam, and Hinduism. The Pew study found that there are 2.2 billion Christians around the world (32% of the world’s population), 1.6 billion Muslims (23% of the world’s population), 1.0 billion Hindus (15% of the world’s population), half a billion Buddhists, and 14 million Jews who follow Judaism [9].

Studies related to religion show that the Muslim population is the fastest growing in the world with an expected rate of 35% in the next 20 years. The global Muslim population is expected to reach 2.2 billion by 2030, more than double the rate of the non-Muslim population. In 2019 alone, the Hajj attracted over 2.5 million pilgrims, while the Kumbh Mela was attended by approximately 150 million people [10]. In addition to creating operational challenges, these large pilgrim numbers are linked with increased vulnerability to infectious diseases. Therefore, this review explores the case study of the Hajj, that is distinguished by a wide range of topics covered by the literature. Further, the Kumbh Mela will also be investigated, the Arba’een pilgrimage, in a similar way to the Hajj, and Christian mass gatherings. The discussion will then summarize the current insights and highlight the significant gaps that are existed in mass gathering healthcare research.

## 2. Materials and Methods

The next sections develop a “traditional or narrative literature review” that is designed to be replicable in the future [11]. The first step of the review involved undertaking a preliminary search of keywords in several electronic databases such as the National Centre for Biology Information, Jstor.org, Google Scholar, PubMed, ScienceDirect, Scopus and the ISI Web of Science. These were chosen because they access complementary datasets and use different research algorithm. The keywords used in this initial search were “mass gathering medicine”, “mass gathering health risks”, and “health risk.” A further and more focused analysis was concentrated adding the name of the case studies to each of the keywords.

As a second step, the abstract sections of the sources identified were analyzed to reveal other relevant keywords that could be associated with the same content. Several new keywords were also identified including “infectious disease risk” and “non-infectious disease risks.” Again, a further and more focused analysis was concentrated adding the name of the case studies to each of the keywords. A clear focus of the literature was concentrated on the three largest religious gatherings, the Hajj, the Kumbh Mela, and the Arba’een pilgrimage, that are reported in the sections below. The final step in conducting the review involved a cross-check of the bibliography in the papers included to identify possible recurrent citations. What emerged was a concentration of the literature on the Hajj event, which will be discussed in more detail. This can be explained by more information being provided by Saudi authorities and the yearly recurrence of the event.

## 3. Results

### 3.1. The Hajj

Hajj is a yearly event that attracts approximately three million Muslims from all over the world, and it represents more than just a physical journey. The annual mass gathering is one of the five pillars under Islam [12]. The primary site that Muslims visit while on the Hajj is the Kaaba. In fact, with the global Muslim population growing every year, and with the fact that Hajj only covers a total of five days, the pressure on Saudi Arabia to accommodate more pilgrims during the Hajj will only increase further. With Islam being the second largest global population, there are millions of pilgrims that wish to embark on the Hajj every year [12]. Consequently, the government controls the inflow of pilgrims using the Hajj visa [13]. That is, it is impossible for one to enter Mecca for the Hajj without a Hajj visa issued solely by the Saudi Ministry of Hajj. These visas are distributed to countries all around the world depending on the proportion of the global Muslim population living in every country. Additionally, the Hajj visa is also one of the primary tools to guarantee public health safety during the mass gathering. All those that are issued with the visa are expected to have first fulfilled a series of preventative health requirements issued by the Saudi Ministry of Health [14]. These requirements are issued before every Hajj cycle and are consequently subject to change depending on the prevailing nature of the public health risk in Mecca. For convenience purposes, travel to Mecca during the Hajj is typically facilitated using travel agencies. This also allows for the easy management of pilgrims as groups, instead of as individuals, once they arrive at Mecca.

#### 3.1.1. Health Risks during the Hajj

The Hajj is unfortunately faced by several high-profile risks. This stems from the fact that this mass gathering has several unique characteristics that creates a conducive environment for health risks. The first of these characteristics is the size of the pilgrim crowds. In fact, elevated population densities of up to nine people per square meter have previously been recorded during the mass gathering [15]. Such congestion is not only scary, but also considerably increases the risk of pilgrims contaminating each other with infections and causing physical harm to each other through stampedes. Unfortunately, the size of the gathering is further complicated by the fact that Mecca covers a relatively small area [12].

The sheer size of the crowds at Hajj greatly stretches the limited public health resources available during the Hajj. It is important to note that the Saudi government avails a lot of public health resources for the mass gathering [16]. In addition to tens of thousands of qualified healthcare professionals and volunteers, dozens of health centers and hospitals are made available during the pilgrimage. For example, during the 2012 pilgrimage, there were a total of 25 hospitals in Mecca and nearby regions, supported by over 140 health centers, used to provide healthcare services to Hajj pilgrims [15]. While these public health resources may seem significant, they are quite few compared to the millions of pilgrims they are meant to serve. As such, in the event of a catastrophic public health incident, available health resources would do very little to reduce the risk of harm to pilgrims.

Another factor that increases the degree of health risk during the Hajj is the climate of Mecca. Saudi Arabia is well known to be a desert nation. As such, it is not surprising that Mecca, one of the country’s interior cities, largely has a semi-arid climate. Even during the winter, temperature levels rarely go significantly low, while in the summer they can hit highs of up to 45 °C [17]. As such, even when the event of Hajj falls in winter months, the climate is not particularly kind to pilgrims. Sadly, recent weather reports seem to indicate that the pilgrimage will, over the course of the next decade, largely fall during the hot summer months in Mecca. In addition to the always-present risk of heat stroke, a hot climate during the Hajj increases the likelihood of fatigue, thus compromising the health of those with weak immunities [17].

Lastly, the diversity of the pilgrim population during the Hajj also increases the level of health risk during the mass gathering. Hajj pilgrims are drawn from all over the world. While this is a fact to be celebrated from a religious standpoint, it creates several complexities from the public health standpoint. Understandably, these pilgrims have widely unique and diverse medical backgrounds. Some originate from regions where certain disease strains are not found in other parts of the world [18]. Additionally, some pilgrims, particularly those from low-income countries, typically have had little-to-no exposure to effective medical care before embarking on the pilgrimage. Given the fact that pilgrims can travel to Mecca within a relatively short period of time, infectious disease that are only unique to certain regions of the world can easily spread at the mass gathering. Further, cultural, and linguistic barriers also prevent some of the pilgrims from accessing immediate and effective care [15].

#### 3.1.2. Infectious-Related Health Risk

Unfortunately, infectious-related health risks are quite common during the Hajj. The spread and dissemination of infectious diseases is highly reliant on close contact and interaction [19]. Owing to its huge crowds, the Hajj provides multiple avenues for the transmission of diseases. In fact, several airborne infections, such as tuberculosis and meningitis, have been recorded at the annual mass pilgrimage [20]. Further, poor hygiene behavior among some of the pilgrims, coupled with inadequate adherence to recommended preventative measures, facilitate the easy spread infectious diseases.

Undoubtedly, the most common infectious diseases during the Hajj are respiratory infections, particularly upper respiratory tract infections. For example, it is reported that up to a third of all pilgrims that embark on the Hajj experience respiratory symptoms associated with the influenza virus [21]. This has consequently led to the influenza infection during the mass pilgrimage to be informally referred to as the Hajj cough. In addition to influenza, adenoviruses, and respiratory syncytial virus (RSVs) are also quite common at the mass pilgrimage. Sadly, there is very little existing research on the epidemiology of upper respiratory tract infections during the Hajj. That said, a 2007 study found that pilgrims that spent a longer time in the Grand Mosque of Mecca had an elevated risk of contracting an acute upper respiratory tract infection [22].

The ease with which upper respiratory tract infections spread during the Hajj has led to the fear that viral pandemic could occur at the mass gathering. In fact, in the last few years, Saudi public health authorities have been forced to implement strict infectious control measures in response to COVID-19, SARS, H1N1, and the MERS Coronavirus [15].

Lower respiratory tract infections, while not as prevalent as upper respiratory tract infections, account for a significant portion of hospital admissions during the Hajj. In fact, as highlighted earlier, pneumonia has been the leading cause of pilgrim hospital admission during several Hajj editions. In 2003, for example, approximately 39% of the pilgrims admitted during the mass gathering were suffering from pneumonia [23]. Additionally, tuberculosis, another lower respiratory tract infection, is often quite common among elderly pilgrims. According to Alzeer [22], in addition to the fact that some pilgrims from tuberculosis endemic countries arrive with the infection while it is in active state, its spread is heavily facilitated by reduced immunity among vulnerable pilgrims owing to fatigue and exhaustion. It is also theorized that the spread of lower respiratory tract infectious disease, particularly tuberculosis, is partly facilitated by the fact that most of the pilgrims travel to Saudi Arabia by air [22]. Long, crowded flights provide a good avenue for the spread of infections.

Infectious gastrointestinal diseases are often also reported during the Hajj. Fortunately, however, these infections do not have particularly high mortality and morbidity rates. Many of the pilgrims that embark on the Hajj in fact experience traveler’s diarrhea [20]. The occurrence of these and other gastrointestinal infections has largely been attributed to poor hygiene standards and food handling. In more serious cases, some pilgrims have been infected by the vibrio cholerae bacteria, leading to a cholera infection. While reported cholera incidents have become quite few in recent years, outbreaks were a common feature at the Hajj before the 1990s [15]. To control the spread of cholera, diarrhea, and other gastrointestinal infections, the Ministry of Health has strict regulations on the importation and handling of food products [14]. Additionally, the ministry also encourages pilgrims to maintain high standards of hygiene throughout their entire visit to Mecca.

Unfortunately, meningococcal disease has also historically also been an infectious health concern during the Hajj. The infectious nature of this disease is typically at an elevated level during the Hajj owing to overcrowding, elevated carrier rates, and high humidity [15]. As a result, there have been several meningococcal disease outbreaks during the Hajj. An outbreak of meningococcal serogroup A was last reported in 1989 [24]. Since then, however, there have been two high profile outbreaks attributed to the serogroup W135. The first occurred in 2000 and affected approximately 1300 pilgrims, while the second occurred in the following year and is estimated to have affected slightly more than 1100 people [25]. Given the high risk to public health associated to meningococcal disease by the Ministry of Health, all pilgrims are required to receive a vaccination before entering Saudi Arabia.

Some of the rituals associated with the Hajj can expose pilgrims to the risk of contracting infectious blood-borne disease. At the end of every Hajj cycle, male pilgrims will typically shave their heads to signify a successfully completed pilgrimage [20]. Unfortunately, however, these haircuts are not always provided in the safest manner. For some of the pilgrims, getting a haircut typically means visiting roadside barbers. These barbers may sometimes use unsterilized blades to cut the hair of multiple people, thus creating an avenue for the possible spread of blood-borne diseases such as HIV and hepatitis [24]. The Ministry of Health recognizes the potential health risks caused by this behavior and consequently encourages pilgrims to receive a hepatitis B vaccination before embarking on the Hajj. Additionally, pilgrims are also advised to be very careful of where and how they receive their haircuts, with only licensed barbers being preferred [24].

More recently, public health officials in and out of Saudi Arabia have become aware of the potential risk for the global spread of antibiotic resistant bacteria through mass gatherings such as the Hajj. Antimicrobial resistance develops when microbial organisms, such as bacteria, survive exposure to an antimicrobial drug, such as an antibiotic, by changing [26]. While gradual resistance to antimicrobial drugs is expected as a result of genetic changes, microbes are becoming increasingly resistant as a result of drug misuse. Antimicrobial resistance is considered a significant global health concern as it effectively makes certain medicines ineffective and increases the risk of infectious diseases being transmitted among people. As such, the concern that a drug resistance globalization could be achieved through the Hajj is valid. In fact, several studies have already proven that antibiotic-resistant bacteria can be transferred and spread by travelers. For example, a study found that a section of travelers returning to the United Kingdom from India acquired New Delhi metallo-beta-lactamase-1 (NDM-1), which has shown significant resistance to almost all beta-lactam antibiotics [27]. Given the fact that the Hajj is not only the largest human mass gathering in the world, but also that it is a recurring event, it creates multiple avenues for the spread of drug-resistant infections through close contact and interaction. Sadly, however, available literature on the patterns of antibiotic use among Hajj-goers, and the actual role they play in the dissemination of antibiotic resistance, is still very limited [26].

#### 3.1.3. Non-Infectious-Related Health Risks

While infectious diseases are by far the most prevalent health risks during the Hajj, they unfortunately are not the only ones. Non-infectious-related health risks occur far less frequently but account for a significantly greater proportion of the mortality during the annual pilgrimage [15]. Sadly, much of the mortality and morbidity associated with non-infectious-related risks are in fact avoidable. As such, it is not surprising that some of the preventative health measures recommended to pilgrims before embarking on the Hajj touch on non-infectious health risks. Given the hot climate in Mecca, it is not surprising that heat-related diseases are a significant health risk during the Hajj. The congestion, difficult terrain, and hot climate experienced during the pilgrimage can sometimes lead to fatigue among pilgrims [15]. However, in extreme cases, high temperatures can also causes heat stroke. For example, a highly deadly mass heatstroke incident was recorded during the 1985 Hajj, in which more than 1000 pilgrims lost their lives [15]. Unfortunately, the sick and the elderly are typically at a higher risk of being affected by heat stroke. Simple recommendations such as using an umbrella, drinking water, and applying sunscreen, can go a long way in effectively preventing the occurrence of heat-related diseases. Additionally, stampedes have been some of the deadliest health risks reported at the Hajj in recent years. Human mass stampedes typically occur when panic occurs within a large crowd leading to uncontrolled mass movement that ultimately leads to injury or death [12]. Many of those killed in mass stampedes die as a result of traumatic asphyxiation [17]. Sadly, given the large crowd densities that are prevalent during the Hajj, scheduling and logistical errors can sometimes result in deadly human stampedes. This was unfortunately the case during the 2015 Hajj pilgrimage, where over 2000 pilgrims lost their lives [12]. Further, before this incident, there had been several other deadly stampede incidents at the Hajj, going as far back as 1990. Though not as prevalent as stampede incidents, pilgrims during the Hajj are also faced with the public health risk of fire incidents. In 1997, a fire incident occurred in the tent city of Mina, claiming the lives of 343 pilgrims and injuring thousands more [12]. The congestion of tents, coupled with the strong winds that blow across the Mina valley, greatly facilitated the spread of the fire. Fortunately, in the years since, strict fire regulations have been introduced that have prevented the recurrence of a similar incident. In addition to tents now being constructed of fire-resistant material, pilgrims are not allowed to cook in their tents [12]. With regards to health risk perception among Hajj pilgrims, several studies have been conducted that have indirectly highlighted the state of health risk perception among Hajj pilgrims [28]. It has been shown that the perceptions and misperceptions that pilgrims have towards health risks and preventative measures greatly influence the uptake of these measures [28]. For example, in a study investigating barriers to vaccinations uptake among Australian Hajj pilgrims, [28] found perceptions that vaccines were unsafe to be one of the biggest barriers. Equally, a study reported that only 44% of pilgrim participants though the health risks at Hajj to be significant enough to seek pre-travel health advice [29]. Unfortunately, however, the number of studies conducted to investigate health risk perception among Hajj pilgrims is too low to draw substantive conclusions.

#### 3.1.4. Preventative Health Measures Recommended by the Ministry of Health

Since 2019, after the outbreak of COVID-19 around the world, the Kingdom of Saudi Arabia has taken strict measures to curb the spread of COVID-19 within the Kingdom. In 2020, the Kingdom cancelled many religious gatherings, halting Umrah ceremonies in early March, halted all international flights, and restricting internal movement [30]. In 2020, the Kingdom also cancelled the Hajj, while the Hajj ceremonies in 2021 returned to a very limited number of pilgrims from within the Kingdom, with the application of social distancing measures and adherence to the muzzle and hand sterilization [31]. Tarawih prayers have been suspended in mosques during the month of Ramadan.

Cognizant of the various health risks that exists at the Hajj, every year the Saudi Ministry of Health publishes an updated list of preventative health guidelines and recommendations that pilgrims are expected to adhere to. The most recently published update was in readiness for the 1439H (2018) Hajj season. Among the diseases of special importance highlighted in the update was yellow fever. The ministry instructed that all pilgrims from yellow fever-endemic countries in Africa and the Americas should show a valid yellow fever vaccination certificate [14]. Additionally, all pilgrims arriving in Saudi Arabia were required to show a valid meningococcal meningitis vaccination certificate. The ministry accepted either the trivalent ACYW135 polysaccharide vaccine or the trivalent ACYW135 conjugate vaccine [14]. For both yellow fever and meningococcal meningitis, pilgrims were required to have received their vaccination shots no longer than 10 days before travelling to the Kingdom. Equally, pilgrims from countries at high risk of polio reintroduction were required to show a poliomyelitis vaccination certificate [14]. The at-risk countries were Afghanistan, Ethiopia, Myanmar, Nigeria, Pakistan, South Sudan, Syria, and Yemen [14].

Further, with regards to seasonal influenza, the ministry did not require all pilgrims to get vaccinated, but rather recommended that they did. It especially advised that persons with compromised immunities, coupled with young children, pregnant women, and the elderly, receive the vaccine. Lastly, pilgrims from countries affected by the Zika virus disease or dengue fever were required to show certification that they have completed disinfection measures [14]. A major concern going into the 2018 Hajj cycle was the growing threat of MERS-CoV and other similar respiratory infections. As such, the ministry set out a series of hygiene recommendations to prevent easy transmission [14]. All pilgrims were advised to wash their hands with soap or a disinfectant after sneezing [14]. This recommendation was also advised after using toilets or before handling food, so as to reduce the risk of contracting a gastrointestinal infection. They were also advised to wear mask and use disposable tissues when coughing or sneezing in crowded areas. Further, pilgrims were advised against consuming any improperly stored or cooked foods, especially meat and milk [14].

Lastly, the Ministry of Health encourages that pilgrims be in good physical condition and actively seek out health education. As stated on numerous occasions, the Hajj can be a very tiring journey, especially considering the hot climate in Mecca. As such, the ministry encourages fellow governments to only grant those pilgrims in the best physical condition to complete the pilgrimage the permission to embark on Hajj [14]. Those with serious health conditions, such as advanced chronic illnesses, are exempt from undertaking the Hajj under Islamic law [14]. Equally, once at the Hajj, pilgrims are encouraged to stay out of direct contact with the sun and drink a lot of water, so as to avoid heat exhaustion. Foreign governments are encouraged to provide pilgrims with basic health education in preparation for the annual mass gathering.

In addition to these preventative measures provided by the Saudi Ministry of Health, several international organizations and governments also provide recommendations for pilgrims. For example, the Centre for Disease Control and Prevention (CDC), publishes a detailed overview of the Hajj pilgrimage on its website, including the various health issues that exist, to prepare American pilgrims [32]. Equally, the WHO regularly issues updates on health issues at the Hajj that pilgrims should be aware of, and the potential measures they can take to better safeguard their health [32]. As such, there are multiple sources through which pilgrims can access information on effective preventative health measures to apply while in Mecca.

#### 3.1.5. Adherence to Recommended Preventative Health Measures

**Figure 1:**
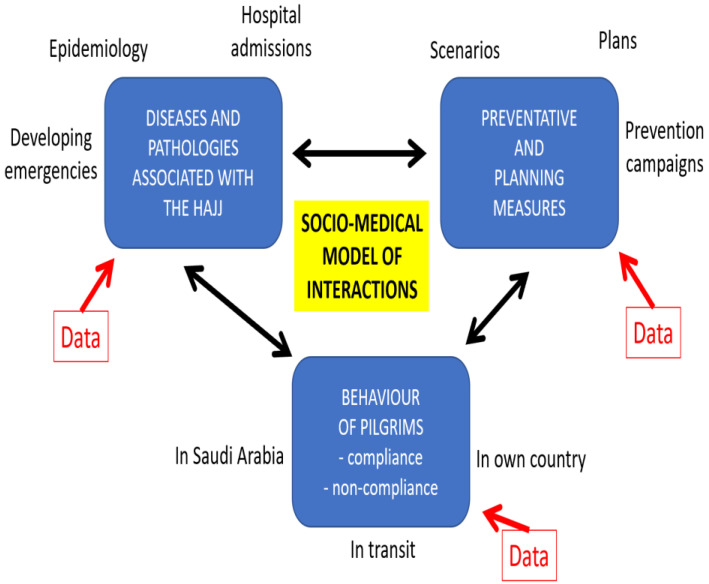
Socio-medical model of how various factors interact to influence health risks at the Hajj.

The socio-medical model of interaction shows that pilgrim behavior also plays a major role in the mitigation of health risks at the Hajj (Figure 1). A significant portion of the studies investigating adherence preventative health measures during the Hajj have concentrated on the uptake of the influenza vaccine. The WHO and numerous national health bodies report vaccination as the most effective mitigation strategy against seasonal influenza [33]. There has, however, been little consensus on the exact rate of uptake among Hajj pilgrims, with results from various studies varying considerably. According to a study by Barasheed et al. [34] on Australian pilgrims, 65% and later 89% of the respondents reported receiving the seasonal influenza vaccine before travelling to Mecca. Another study on Malaysian pilgrims reported that approximately 73% of the pilgrims had received vaccine [35]. The uptake rate is, however, considerably lower in other studies. A 2013 study on French pilgrims found that none of the 129 respondents had received the seasonal influenza vaccine before embarking on the pilgrimage [36]. Additionally, the uptake of the vaccine was found to be equally varied among healthcare professionals working during Hajj, with only 5.9% of the respondents having received the vaccine [37]. Even a study investigating the uptake of the influenza H1N1 vaccine before the 2009 Hajj found that only 30% of the pilgrims had received it, despite the publicized concerns about a global pandemic [20]. Awareness of other preventative measures targeting infectious respiratory diseases, such as good hygiene and face masks, was also concerning, with only 50% of the respondents stating that they knew about it [20]. The uptake of face masks among pilgrims has been found to be equally varied in studies, ranging from 0.02% to 98% [34]; however, the reported uptake averages to about 50% [34].

Arguably the greatest success experienced concerning adherence to recommended preventative health measures has been with the uptake of the meningococcal disease vaccine. According to Memish et al. [38], the compliance rate for the Ministry of Health directive on meningococcal disease stands at a respectable 98.2%. Equally, the reported uptake rate for Hajj healthcare workers was reported at 82.4%, specifically for the quadrivalent (ACYW135) vaccine [37]. This high uptake rate perhaps explains why there have been no reported meningococcal disease outbreaks in recent years, while the reported incidents of some other infectious diseases has remained high. For example, the uptake of the pneumococcal has consistently been reported at below 30%, despite pneumonia being one of the leading causes for hospital admissions and intensive care unit visits during the Hajj [39].

### 3.2. Kumbh Mela

Kumbh Mela is a mass gathering held in India. As with the Hajj, the Kumbh Mela is a religious mass gathering. However, it is based on Hindu mythology as opposed to Islam [40]. Its mythological background stems from a belief that nectar with the ability to create immortality was poured at four different locations within India. Every three years, the Kumbh Mela draws Hindu pilgrims from all over the world to one of these four locations. These four locations are on the banks of the River Ganga, River Godavari, River Kshipra and at Triveni [40]. Unlike the Hajj, the exact duration of the Kumbh Mela and the exact number of rituals can differ significant from edition to edition. For example, during the 2013 edition, the mass gathering lasted close to two months [40]. Additionally, there were a total of six specified bathing days. However, participants in the Kumbh Mela are not required to stay for the entirety of the mass gathering, and neither are they required to take a dip in the river. The Kumbh Mela event attracts close to 100 million participants and is also faced by a wealth of health challenges [40]. However, owing to the strong commitment of public health authorities in India, the Kumbh Mela has not had significant disaster events. A major reason for this is the fact that a greater portion of the event’s budget has been put towards health, an increase from 20% in 1966 to 45% in 2013 [41]. There has also been an increased reliance on the collection and analysis of health data to inform relevant preventative measures. Equally, telemedicine and teleconsultation services have been introduced to enable the treatment of complex conditions and cases in Kumbh Mela from tertiary hospitals located hundreds of miles away. Further, in recent years at Kumbh Mela there has been a move away from compulsory inoculation in support of improved sanitation and water services and increasing access to quality medical services.

#### 3.2.1. Health Risks during the Kumbh Mela

There are several factors in the Kumbh Mela that make the mass gathering susceptible to health risks. Arguably the greatest vulnerability is the large number of participants. At approximately 100 million participants, the Kumbh Mela is approximately 30 times larger than the Hajj [42]. Additionally, many of those who attend the Kumbh Mela are from low socio-economic backgrounds with unknown medical backgrounds. For example, it is estimated that up to 56% of all children in India aged between one and two years old are not sufficiently immunized [40]. Further, the fact that this mass gathering is held at four different positions, all of which are the banks or rivers, has complicated the creation of permanent facilitative infrastructure. Instead, a Mela city is constructed at one of the chosen sites several weeks before the commencement of the mas gathering. However, some of the supporting infrastructure in this city is not always sufficient to support the tens of millions of visitors. Equally, unlike the Hajj where almost all the pilgrims arrive by airplane, Kumbh Mela participants arrive by train, road, and foot [41]. This diversity in entry methods not only makes it difficult for authority to track visitor’s movements, but also to undertake effective health surveillance. Further, depending on the duration of the Kumbh Mela, it can sometimes coincide with winter months, thus exposing millions to extremely low temperatures.

#### 3.2.2. Infectious Disease Risk

After 2019, and after COVID-19 entered India in early 2020, the virus spread across India like wildfire, due to India’s overpopulation, disparity in education level, and low income. Large numbers of infections were recorded, despite the increasing efforts to follow measures to protect against the virus, such as maintaining social distancing, wearing masks, and using hand sanitizers [43].

Cholera is the most prevalent infectious disease risk at the Kumbh Mela. During this mass gathering event, millions repeatedly and concurrently take holy baths in the river. Some of the participants also relieve themselves in the water, while others drink the same river waters. As such, should the water be contaminated, millions can easily become infected. A high number of bathers, coupled with hot weather, facilitates the growth of vibrio cholerae [40]. Additionally, the situation is further complicated by the fact that cooking is neither regulated nor supervised. Rather than prepare their own food, many pilgrims instead purchase cheap meals from the surrounding communities [40]. Further, negative sanitation practices, such as open defecation, is still widely practiced at the mass gathering event. As such, there are multiple avenues through which visitors can encounter the cholera pathogen.

Other infectious disease that are spread through contaminated water are also prevalent at the Kumbh Mela. While deadly cholera outbreaks were common before the mid-20th century, no outbreaks have been reported since [41]. However, the event has not experienced similar success with diarrhea. According to Balsari et al. [44], the incidence rate of diarrhea at the Kumbh Mela stands at 5%. The same study found that most diarrhea cases were reported approximately 48 h after the designated bathing date. As such, the drinking of river waters can be inferred as the main avenue for the spread of diarrhea at the mass gathering event.

While infectious respiratory diseases are not linked with significant mortality at the Kumbh Mela, they are still very prevalent. Overcrowding, stress, and exhaustion, facilitate the spread of upper respiratory tract infections such as influenza. Additionally, the use of wood fuel and cow dung to create fires has caused many to seek treatment for persistent coughs. In fact, approximately 23% of the 15,000 patients treated during the event received treatment for cough-related respiratory infections during the 2013 Kumbh Mela edition [45]. Further, owing to the fact that tuberculosis is endemic to India, there is always a risk of the disease being spread at the annual mass gathering.

Further, some of the rituals undertaken at the Kumbh Mela expose some visitors to blood-borne diseases. Some visitors undergo initiation to become sadhus (monks) at the mass gathering. While many of the rituals involved in this initiation are not shown openly, the initiates are required to shave their heads [40]. Given that this clean shave is often with a knife or blade as opposed to sterilized equipment creates a risk of the spread of blood-borne diseases. Some of these diseases may include hepatitis and HIV/AIDS; that said, there is little research into the spread of these diseases at Kumbh Mela rituals [40].

#### 3.2.3. Non-Infectious Disease Risk

As with the Hajj, the Kumbh Mela is also faced by a significant risk of human stampedes. While this mass gathering does not occur within a fixed structural boundary, the movement of pilgrim is mainly limited to the banks of rivers [44]. As such, overcrowding is common on these riverbanks, and in extreme cases has resulted in deadly stampede events. For example, in 1954 a mass stampede occurred at the mass gathering killing 500 people and injuring thousands more [44]. While there have not been other significant deadly mass stampedes at Kumbh Mela sites, several have occurred at facilitative venues and sites. For example, in 2013, 36 people lost their lives at a railway station as visitors struggled to board trains headed to Kumbh Mela [40]. The occurrence of sectoral conflict at the Kumbh Mela has also been directly and indirectly linked with injuries and deaths. As intimated earlier, this mass gathering attracts people from all walks of life. Some of the visitors belong to different religious sects. Since some of these sects are not cordial with each other, the Indian government has in the past tried to schedule different bath times for these groups. However, several clashes have still been reported between rivalling sects. For example, an attempt by one sect in 2010 to flex its might by driving into a crowd created a stampede that injured several persons [44].

#### 3.2.4. Preventative Health Measures Recommended by the Ministry of Health

Regarding the COVID-19 pandemic, the Indian government followed several measures to contain the spread of the virus; a nationwide lockdown was imposed at the beginning of 2020. India succeeded in reducing the number of cases at that time but depending on the economic and social conditions in India, the lockdown was gradually eased, which pushed the number of injuries rapidly upwards in the middle of 2020. Consequently, religious gatherings such as the Kumbh Mela are creating a major public health risk with a catastrophic increase in COVID-19 cases. Consequently, different dates for bathing/toothing were announced and the upper body of the grooves showed some willingness to hold the Kumbh Mela in a restricted manner. It was recommended that the number of participants in the Kumbh Mela be restricted in 2021 [43].

In recent years, due to the strong commitment of the public health authorities in India, the Kumbh Hela has not had a significant accident [40]. There have been different reasons for this: the fact that a greater portion of the event’s budget has been put towards health, an increase from 20% in 1966 to 45% in 2013 [41]. There has also been an increased reliance on the collection and analysis of health data to inform relevant preventative measures. Equally, telemedicine and teleconsultation services have been introduced to enable the treatment of complex conditions and cases in Kumbh Mela from tertiary hospitals located hundreds of miles away. Further, there has also been a move away from compulsory inoculation in support of improved sanitation and water services and increasing access to quality medical services. Visitors are advised not to drink river waters, but instead consume the piped water provided by the government in the temporary Mela city [44]. Equally, they are also advised to handle and store food in a sanitary manner.

#### 3.2.5. Adherence to Recommended Preventative Health Measures

The current literature shows very little research into the adherence preventative health measures by governmental authorities. That said, some studies have shown that despite government insistence against it, many pilgrims continue to drink water from the rivers as they consider it is holy [40]. Additionally, many insist on drinking due to superstitious beliefs about alternative water sources, such as wells. Further, the lack of adequate enforcement means that many pilgrims do not adhere to food-related preventative measures [40].

### 3.3. Arba’een Pilgrimage

The annual Arba’een pilgrimage is held every year in Karbala, Iraq, and challenges the Hajj in size [46]. The mass gathering attracts approximately 20 million Shia Muslim pilgrims annually. This large number has thus far not only created logistical challenge, but also public health challenges. However, this event is distinguished by the limited literature available and focused on the direct healthcare impacts.

These challenges at the annual mass gathering event are partially caused by the defects of the health infrastructure in Iraq. Years of war have left large sections of the country destroyed, including many that touch on the healthcare system [46]. Additionally, organizers cannot adequately rely on the health infrastructure in the country when preparing for the annual event.

Despite the unavailability of significant research evidence, the Arba’een pilgrimage is believed to have infectious disease risks. According to Karampourian et al. [46], this largely stems from the fact there have been very little effort to address the underlying factors that determine the prevalence of infectious diseases. In addition to insufficient public health facilities, the Arba’een pilgrimage is also characterized by insufficient sanitation facilities. Additionally, little regard is given to the diversity and density of the population that embarks on the pilgrimage. Owing to these complications in the control of underlying factors in the prevalence of infectious diseases, a wealth of infectious diseases could affect pilgrims at the annual mass gathering event.

Research has also shown the level of risk perception among Arba’een pilgrimage to be worryingly low. Many of those who embark on the pilgrimage do not have sufficient knowledge on public and private health. As a result, many continue to engage in risky health behaviors such as unhygienic beliefs and the refusal to take medication [40]. This low awareness among pilgrims propagates the false idea that they will not be exposed to any health risks during the annual mass gathering event. Consequently, disregarding health advice and requirements is seen as a harmless act.

### 3.4. Christian Mass Gatherings

Religious gatherings for Christian groups include World Youth Day, which is held every two or three years in the Catholic Church, and nearly a quarter of a million Catholic youths in different cities attend an event hosted by the Pope. For example, the closing mass for World Youth Day in 1995 attracted 5 million participants in the Philippines. In 2015, 6 million people also gathered for World Youth Day in the Philippines, led by Pope Francis [47].

The Christian pilgrimage in France, the most famous, attracts more than 5 million in the city of Lourdes, and it is a year-round event. Lourdes, a small city in the southern Pyrenees, is the second most visited French city after Paris. It is the third most important pilgrimage site in the world of Catholics after the Holy Land and Rome [48]. Because most of the religious gatherings of the Christian community are held in developed countries, the infrastructure of these countries helps reduce the problems of overcrowding and is based on developing strategies and protocols to manage the gatherings properly. Like any other grouping, communicable and non-communicable diseases can be found among people. Most countries, such as France and Italy, have successfully managed religious congregations. Despite this, history records some cases of contagious diseases due to religious gatherings in churches. In 2005, in the state of Indiana in the United States of America, an outbreak of measles among unvaccinated children occurred after a case of an unvaccinated girl was recorded, after a church gathering of 500 in the state [49].

Now, with the occurrence of the COVID-19 pandemic, most countries have gone on to impose closures and bans for certain periods between 2019 until now, to limit the spread of the virus. It also limited the number of people in religious gatherings with the imposition of social distancing and the use of protection methods such as wearing masks and gloves and frequent sterilization. In St. Peter’s Basilica (Vatican), Pope Francis’ Easter Sunday mass was held in April 2020 without the audience and broadcast live on TV (The Straits Times, 2020). Additionally, Protestant churches around the world held Easter through live broadcasts without church attendance. Most places of worship in the world have suspended physical worship and regular prayers and replaced their services to internet broadcasting [49].

## 4. Discussion

This review showed that public health risks during religious and mass gatherings have been investigated extensively, as to the pre-travel requirements, but there are still some evident gaps in these studies. It can be noted much of the current research has focused on the Hajj and some insights from other mass gatherings have been overlooked, as well as other causes that affect infectious diseases in mass groups. This may be due to the fact that Hajj is an annual routine that happens every once in a while, and because most of the world’s population embrace religions, and finally because Hajj is linked to a specific place to perform it and therefore includes travel and climate and environmental change, which may cause diseases, or mutations of viruses due to mixing people from different parts of the world.

One of the main causes is the strong evidence of climate change studies, which are showing disease emergence from land and climate change that are affecting mass groups with emerging diseases. Emergence or resurgence of numerous infectious diseases are strongly influenced by environmental factors such as climate or land use change [50]. As the world grows warmer and various infections emerge, this affects massive groups and how these diseases can be countermanded [50]. Besides climate changes, crowd sizes are also affecting major cities and mass gathering within these cities.

Half of the population live in large cities, with 8.9 million people in London, U.K. In the 9th largest section of London is Notting Hill Carnival, in central London, with one million people gathering in just two days, in an area that already has over 750,000 thousand people living there [51]. This type of urbanization creates massive congestion, similar to the Hajj and other specific religious and sport groups, which is now concentrated within the heart of every major city. More studies need to be carried out on mass gathering medicine and how it can be generalized from mass gatherings, such as the Hajj, to everyday life in any major city, refugees in camps, and any other mass group of people.

A common gap in all the case studies analyzed is the limited evidence associated with risk. Indeed, the literature on disaster reduction suggests considering this element as a critical component to address vulnerabilities in emergency planning [1]. In other words, it can be noted that in all case studies the uptake of preventative measures is not always the same for all travelers [40], but this is not supported by the discussion on the extent to which pilgrims are aware of health risks. The same concerns are with any mass groups, where groups choose to ignore the warnings for vaccinations or other preventive steps against infectious diseases.

Even in the Hajj, much of the research has concentrated on the causes and consequences of infectious disease risks. Much less, evidence has not been commensurate in looking into how pilgrims perceive health risks at mass gatherings and specifically the Hajj. Johansson et al. have stated the concern is a macroscopic (crowd) level, rather than an individual level, or a microscopic level [51]. In other words, such things that could be restricting factors, such as immunizations, screening, quarantine, and travel restrictions are on an individual basis; but the solution is not an individual problem, but a mass crowd or a macroscopic concern [51].

This is common with the analyses developed on the Kumbh Mela, where numerous inferences have been made about the behaviors of pilgrims without a commensurate look into how they perceive health risks [52]. As highlighted above, in this case, people’s decisions on the adoption of preventative health measures are greatly dependent on how they perceive the underlying health risks [52]. During the Notting Hill Carnival, health authorities need to consider basic human needs, including portable waste containers, proper food containers, potable water, etc. [51]. Finally, in all cases, the literature overlooked the demographics and cultural drivers that could influence risk perception, risk awareness, and the adoption of preventative measures.

These gaps suggest focusing on the current state of art on the hazard, exposure, and physical vulnerability components of disaster risk. Instead, it is missing the understanding of vulnerability as a process where environmental, social, and economic factors interact dynamically [53]. In our cases, for example, physical vulnerabilities could manifest in the lack of adequate sanitation [41]. However, the inability to communicate with health professional at a mass gathering due to cultural or linguistic differences, as well as in policies and practices, can be seen as a barrier that increase disaster risk by increasing the vulnerability of pilgrims. In other words, the lack of communication or negligence in addressing the underlying factors of vulnerability can act as an amplifying factor of risk, which could be counteracted by developing an emergency planning process supported by positive factors such as governance [53].

While there are numerous models that attempt to explain the differential use of preventive behavior, one of the most consistently and widely used is the health belief model (HBM) [53]. This model holds that individuals base their decisions on preventative behaviors based on two key elements. The first element is their believed susceptibility to the health risk at hand [52]. For example, a healthy young pilgrim may feel that their chances of contracting influenza while on the pilgrimage are quite low. The second element is the perceived severity of the health risk at hand [52]. For example, the same pilgrim may feel like even in the event that they contract seasonal influenza, the disease is not too serious. Therefore, based on the combination of these two trains of thought, the pilgrim may decide to forego seeking additional health information on influenza and taking up the preventative behaviors recommended by the Saudi Ministry of Health. However, the HBM goes on to clarify that these two elements are greatly influenced by information. Information can create an increased level of threat perception, but that alone is typically not sufficient to pressure an individual into adopting preventative behavior. Instead, the decision whether to act or not is determined by a further two factors. The first factor is the overall perceived benefit of the proposed preventative behavior. Secondly, the individual will typically assess the barriers associated with the preventative behavior at hand, such as cost or embarrassment [52]. The eventual balance between the perceived benefits of a preventative action, compared to its costs, ultimately determine whether an individual adopts it or not. Just as with the HBM, pilgrims at the Hajj typically make decisions subjectively, thus explaining why despite factual evidence for the need of preventative health measures, some still choose not to adopt them. Understanding how people perceive health risks is a necessary ingredient in also understanding the preventative health measures people choose. Equally, how Hajj pilgrims perceive health risks at Mecca plays a central role in informing whether or not they undertake pre-travel health advice-seeking behavior and/or adopt the preventative health measures recommended by the Ministry of Health. This is an example of HBM that is widely used as preventive behavior to reduce the risk. Further, this acceptability by pilgrims would help decrease the burden of chronic lung disease, leading to better quality of life [54,55,56].

Several psychological outcomes are associated with mass gatherings, which include empowerment, mood improvement, and self-esteem [57]. In turn, several processes are associated with mass gatherings which include sharing emotional experience and shared identity. Participation in group activities improves the wellbeing of members, while the connection between the participants helps to provide much needed social and psychological support [57]. In addition, water, sanitation, and hygiene measures at all mass gatherings may benefit from increasing strategic planning and implementation techniques to decrease risk of morbidity and death from infectious agents that grow in high population density [58].

**Figure 2:**
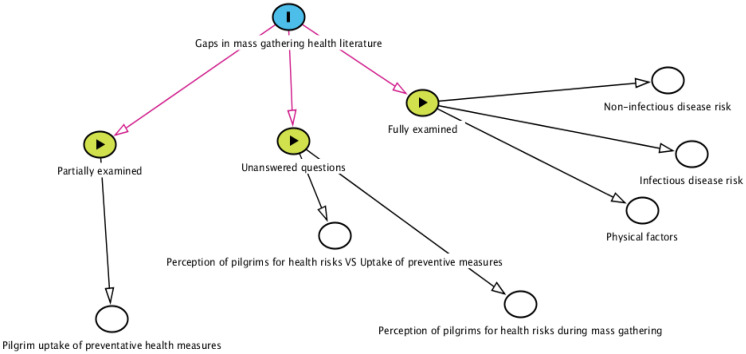
Gaps in current mass gathering health literature.

In conclusion, Figure 2 summarizes the findings of this review, highlighting the differences between factors that have been investigated, partially investigated, and open questions for future research. The next section will drive the conclusions of this review, highlighting new areas for future research.

## 5. Conclusions

The literature on crowd medicine has developed in recent years, analyzing various approaches to mitigating health risks. However, current research has largely focused on the epidemiological and physical conditions that influence these health risks, such as the risks of infectious diseases. This state-of-the-art review highlighted the health risks of religious congregations. Due to the importance of religion, and the presence of large numbers of people belonging to different religious sects, mass religious gatherings are frequent in the Christian, Islamic, Hindu, and other worlds. These gatherings include the international movement of pilgrims, which has proven the danger of the globalization of infectious diseases, especially COVID-19, in addition to the introduction of viruses, and drug-resistant bacteria.

However, there is insufficient evidence on how pilgrims perceive risks, the factors that influence the dissemination of information, and how these elements affect preparedness and training. However, most countries that witness religious gatherings (such as France, the Vatican, and the Kingdom of Saudi Arabia), impose strategies to limit the spread of diseases and injuries, and use monitoring and regulatory protocols, to make these gatherings safer. During the years 2020 and 2021, with the outbreak of COVID-19, most countries of the world imposed strict measures to limit the spread of the virus, including mass religious gatherings.

These measures ranged from stopping the gathering and replacing it with methods of broadcasting via the Internet and television, or restricting the number of people in the gathering with the application of social distancing and the use of masks and hand sterilization. Despite the recommendations contained in documents such as the SFDRR, countries in the third world still do not provide a satisfactory level in managing religious gatherings and mitigating their health risks. Thus, the social and environmental drivers associated with health risks are not well understood in their effects on vulnerability mitigation and risk reduction in general. In other words, future research will benefit by optimizing the context in the current framework for disaster reduction, to address potential drivers and root causes. Finally, we hope that mass gathering regulators in general will use new methods for disease control and prevention, using new digital technologies to detect risks. 

## Data Availability

Not applicable.
